# Reticular Basement Membrane Remodelling Regulates Bronchial Epithelial Attachment, Barrier Integrity and Inflammatory Signalling in Asthma

**DOI:** 10.3390/arm94030038

**Published:** 2026-06-10

**Authors:** Aileen Hsieh, Jenna Barker-Mulleder, Chen Xi Yang, May Fouadi, Tillie-Louise Hackett

**Affiliations:** 1 Centre for Heart Lung Innovation, St. Paul’s Hospital, Vancouver, BC V6Z 1Y6, Canada; aileen.hsieh@hli.ubc.ca (A.H.); jennabm@shaw.ca (J.B.-M.); yolanda.yang@hli.ubc.ca (C.X.Y.); may.fouadi@hli.ubc.ca (M.F.); 2 Department of Anesthesiology, Pharmacology and Therapeutics, The University of British Columbia, Vancouver, BC V6T 1Z3, Canada

**Keywords:** bronchial epithelial cells, reticular basement membrane, extracellular matrix, airway remodelling, asthma, collagen, TSLP

## Abstract

**Highlights:**

**What are the main findings?**

- Extracellular matrix (ECM) proteins enriched in the remodelled asthmatic reticular basement membrane (RBM) (collagen-I, -III and fibronectin) accelerate epithelial attachment, spreading and barrier formation in primary bronchial epithelial cells.
- Fibrillar collagen-I and -III selectively increase epithelial alarmin TSLP (thymic stromal lymphopoietin) secretion without inducing a broad pro-inflammatory cytokine response.

**What are the implications of the main findings?**

- RBM ECM remodelling in asthma is not solely a structural feature but functions as an active regulator of epithelial repair, while simultaneously inducing alarmin TSLP signalling.
- RBM remodelling may promote the persistent epithelial alarmin TSLP signalling observed in asthmatic patients, highlighting the need for targeted biologics.

**Abstract:**

Asthma is characterized by persistent airway epithelial dysfunction and remodelling of the reticular basement membrane (RBM). In healthy airways, the RBM is primarily composed of the extracellular matrix (ECM) proteins laminin and collagen-IV, but in remodelled asthmatic airways, the RBM has increased deposition of collagen-I, -III and fibronectin. Here, we systematically compared the effects of collagen-I, -III, -IV, fibronectin, laminin, and bovine serum albumin (BSA) control on bronchial epithelial cells (BECs) from six healthy controls and seven individuals with asthma. Epithelial attachment, spreading and barrier function were assessed in real time over 72 h using electrical cell–substrate impedance sensing. Cell culture supernatants were analyzed for release of epithelial cytokines, thymic stromal lymphopoietin (TSLP), interleukin (IL)-6, IL-8, and IL-11 using ELISA. BECs from both control and asthma donors had faster cell attachment, spreading, and barrier formation on collagen-I, -III, -IV, and fibronectin compared to laminin and BSA. BECs from both control and asthma donors cultured on collagen -I and -III produced more TSLP, but had no effect on IL-6, IL-8, and IL-11 expression. In summary, remodelling of the RBM in asthma may promote epithelial barrier formation whilst simultaneously enhancing epithelial-derived Th2 inflammation through increased TSLP release.

## 1. Introduction

Asthma is a chronic inflammatory disease of the conducting airways characterized by airway remodelling, including thickening and dysfunction of the airway epithelium, reticular basement membrane (RBM), and underlying mesenchyme with increased angiogenesis, fibroblast number, and smooth muscle mass [1,2,3]. Within the airway wall, the bronchial epithelium forms the primary interface to the inhaled environment and, in response to inhaled allergens or pathogens, regulates tissue repair through bidirectional communication with the underlying mesenchymal cells and recruitment of inflammatory cells [4,5,6,7,8]. Disruption of epithelial barrier integrity is a key feature of asthma and is thought to contribute to persistent airway inflammation and remodelling [7,9,10].

The bronchial epithelium is pseudostratified and composed of three main cell types, mucus-secreting, ciliated, and basal cells. While all airway epithelial cells interface with the RBM, basal cells play a central role in maintaining epithelial integrity by anchoring the epithelium to the RBM via hemidesmosomes and providing structural support for more superficial cells through desmosomal connections [11,12]. Basal cells also serve as the primary progenitor population, which become activated to migrate, proliferate, and regenerate the epithelium after injury. In asthma, basal cell numbers are increased [13], and epithelial barrier integrity is compromised, with disruption of intercellular junctional complexes, including tight junction, adherens junction, desmosomal, and gap junction proteins [14,15,16,17].

In response to environmental exposures such as allergens, pollutants, and pathogens that induce epithelial injury, the bronchial epithelium releases alarmins and cytokines that initiate and amplify the immune response. Thymic stromal lymphopoietin (TSLP) is a key epithelial-derived alarmin upregulated in asthma that promotes type 2 inflammation through activation of dendritic cells [18], innate lymphoid cells [19], mast cells [20], basophils [21], eosinophils [22], and CD4+ T cells [23]. In addition, bronchial epithelial cells produce inflammatory cytokines such as interleukin (IL)-6, IL-8, and IL-11 [14,24,25], which contribute to innate immune cell recruitment and tissue repair through mesenchymal activation [25]. During bronchial epithelial repair, cells initially migrate over a provisional fibronectin-rich matrix, after which mesenchymal fibroblasts deposit fibrillar collagen-I and -III to stabilize the repairing tissue and support epithelial cell proliferation and differentiation [26,27,28]. In asthma, the RBM is remodelled, with increased deposition of fibronectin and collagen-I and -III, in addition to the native basement membrane components collagen-IV and laminin [29,30,31,32,33]. While the RBM provides structural support and regulates growth factor availability, the functional consequences of these ECM compositional changes on epithelial barrier function and cytokine release remain poorly defined.

In this study, we investigated how ECM proteins associated with tissue repair and RBM remodelling (collagen-I, collagen-III, and fibronectin) compare with native basement membrane components (collagen-IV and laminin) in regulating basal epithelial cell attachment, spreading, and barrier formation. This was achieved using primary basal bronchial epithelial cells from control and asthmatic donors in combination with electrical cell–substrate impedance sensing (ECIS), a label-free impedance-based platform that enables longitudinal assessment of epithelial barrier properties at sub-nanometre resolution [34,35]. We further investigated whether distinct ECM proteins influence the release of epithelial alarmins, including TSLP and repair cytokines IL-6, IL-8 and IL-11.

## 2. Materials and Methods

### 2.1. Cell Culture

Primary bronchial epithelial cells were obtained from 6 healthy control donors with no respiratory disease and 7 individuals with asthma who were diagnosed with asthma since early childhood (Table 1). The lungs donated for research were obtained through the International Institute for the Advancement of Medicine. Bronchial epithelial cells were isolated from the proximal conducting airways using pronase digestion as previously described [13]. The lungs were also inflated and frozen, then assessed for airway remodelling of the basement membrane using histology as previously described [36,37,38]. The bronchial epithelial cells were expanded in Bronchial Epithelial Growth Medium (BEGM; CC-3170 Lonza, Basal, Switzerland), supplemented with SingleQuot™ additives, including bovine pituitary extract, hydrocortisone, insulin, transferrin, retinoic acid, epinephrine, triiodothyronine, and gentamicin/amphotericin-B in a humidified incubator at 37 °C and 5% CO_2_. All bronchial epithelial cells were used between passages 4 and 5. The cell culture experiments were conducted with institutional approval from the University of British Columbia’s Providence Research Ethics board #H13-02173.

### 2.2. Epithelial Barrier Function

Electric cell–substrate impedance sensing (ECIS, Applied Biophysics Inc., Troy, NY, USA) was used to monitor real-time barrier function as previously described [39]. Briefly, eight well chamber slides containing 40 electrodes per well (8W10+ arrays, Applied Biophysics Inc., Troy, NY, USA) were coated for 1 h with 50 µg/mL of the following matrices: collagen-I (Rat Tail, Natural, 354236, Corning^®^, Corning, NY, USA), collagen-III (Human, 5021, Advanced Biomatrix, Carlsbad, CA, USA), collagen-IV (Mouse, Natural, 354233, Corning^®^, Corning, NY, USA), laminin (Mouse, Natural, 354232, Corning^®^, Corning, NY, USA), and fibronectin (Human, Natural, 354008, Corning^®^, Corning, NY, USA) or bovine serum albumin (BSA, A9418, Sigma-Aldrich, St. Louis, MO, USA). The arrays were then washed with Bronchial Epithelial Basal Medium (BEBM, Lonza, 3170, Basel, Switzerland), and bronchial epithelial cells were seeded in duplicate in ECIS arrays at a density of 100 K cells per well. Experiments were conducted for 72 h, during which resistance and capacitance data were recorded 14–17 times per hour and normalized to cell-free electrode measurements at time zero (*n*/*n*_0_). The cell culture medium was refreshed at 48 h.

### 2.3. Cytokine Measurements

Cell-free tissue culture supernatants were harvested at 48 h post-treatment and analyzed in duplicate using Duo Enzyme-link Immunosorbent Assay (ELISA) kits for interleukin-6 (IL-6, DY206), interleukin-8 (IL-8, DY208), interleukin-11 (IL-11, DY218) and thymic stromal lymphopoietin (TSLP, DY1398, all from R&D Systems, Minneapolis, MN, USA). The assays were performed according to the manufacturer’s instructions. All cell culture supernatants were run without dilution on the ELISAs. The assays were read at 450 nm with a reference wavelength of 570 nm using the Spectramax iD3 plate reader (Molecular Devices, San Jose, CA, USA), and the SoftMax Pro 7 software (Molecular Devices, San Jose, CA, USA) was used to generate the standard curves.

### 2.4. Lactate Dehydrogenase Assay

Cell-free supernatants were used to determine cell viability following incubation on the different ECM proteins. Lactate dehydrogenase (LDH) release by bronchial epithelial cells was measured with an LDH assay kit (Cat: ab65393, Abcam, Cambridge, UK) with a standard curve of recombinant human lactate dehydrogenase protein (Cat. ab93699 Abcam, Cambridge, UK) according to the manufacturer’s protocol. The Spectramax iD3 (Molecular Devices, San Jose, CA, USA) was used for colorimetric detection of LDH protein.

### 2.5. Statistical Analysis

ELISA data from independent experiments were assessed using non-parametric Kruskal–Wallis tests followed by Dunn’s post hoc multiple comparisons test in Graph Pad Prism^®^ 10 (GraphPad Software, Inc., San Diego, CA, USA). For the ECIS time-series data, a one-phase decay model was used to analyze capacitance data (64 kHz), and a one-phase association model was used to analyze resistance data (500 Hz) in Graph Pad Prism^®^ version 10. From these models, half-life (the time required to achieve half-maximal decline/increase in capacitance/resistance of cells growing on different matrices) and the spreading rate constant (*K*, 1/Hr) of these cells) were calculated. The half-life and rate constant were further analyzed for statistical differences using non-parametric Kruskal–Wallis followed by the two-stage step-up method of the Benjamini, Krieger and Yekutieli post hoc test.

To assess differences in normalized capacitance and resistance of different coatings over time, we applied a generalized additive mixed (GAM) model. This approach has a random effect component to take into consideration multiple measures per replicate in the longitudinal ECIS data. The model structure was specified as:

Capacitance or resistance ~ coating+s(time)+ s(time:coating)+(1|Replicate)

We were able to test (1) coating effects (differences in time-averaged normalized capacitance/resistance between ECM coatings), (2) time effects (changes in normalized capacitance/resistance over time), and (3) the coating-by-time interaction (differences in temporal trajectories between coatings). To compare between control and asthma groups, the model below was used to model the data:

Capacitance or resistance ~ Disease+s(time)+ s(time:Disease)+(1|Replicate)

All the modelling was performed using the R package ‘mgcv’ in the R statistical computing environment (version 4.3.0). For GAMM analyses, statistical significance was determined using *p* values derived from the model fits, and multiple comparisons were adjusted using the false discovery rate (FDR) where applicable. A threshold of *p* < 0.05 was considered statistically significant.

## 3. Results

### 3.1. Basal Bronchial Epithelial Cells Attach More Rapidly on Collagen-I

**Figure 1:**
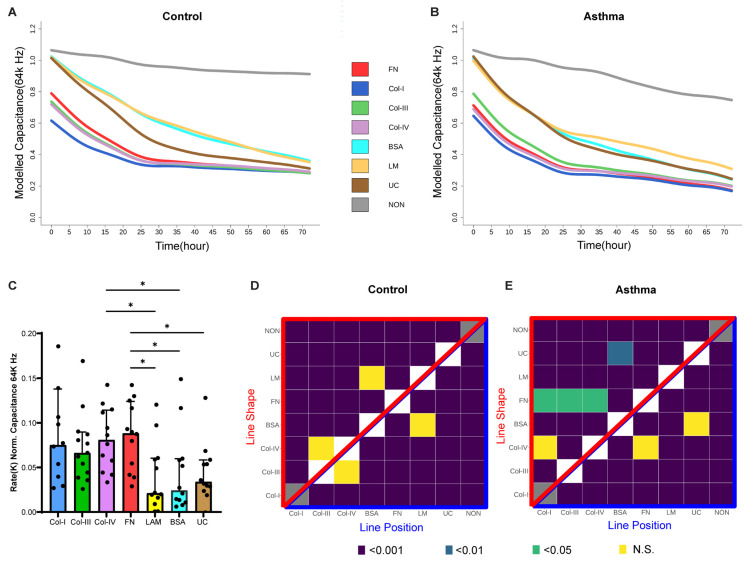
Basal bronchial epithelial cell attachment on different RBM extracellular matrix proteins. Basal bronchial epithelial cells from control (*n* = 6) and asthma (*n* = 7) donor lungs were grown on ECIS electrodes coated with 50 uL/mL of fibronectin (FN, red), collagen-I (Col-1, blue), collagen-III (Col-III, green), collagen-IV (Col-IV, purple), bovine serum albumin (BSA, turquoise), or laminin (LAM, yellow) and compared to uncoated electrodes (UC, brown) or electrodes with no cells (NC, grey). (**A**,**B**) Generalized additive mixed model (GAMM) curves showing normalized 64 kHz capacitance over time for control (**A**) and asthma (**B**) epithelial cells across seven ECM substrates. (**C**) Rate constant (K) was calculated using a one-phase decay model; higher values indicate faster attachment. A Kruskal–Wallis test with the two-stage step-up method of Benjamini, Krieger and Yekutieli post hoc was used. (**D**,**E**) Heatmaps of BH-FDR-adjusted *p*-values from pairwise comparisons of GAM-modelled capacitance between ECM conditions for control (**D**) and asthma (**E**). The upper triangle (red) represents differences in curve shape (group-by-time interaction), while the lower triangle (blue) reflects average capacitance. * *p* < 0.05. *p* < 0.05 = green; *p* < 0.01 = blue; *p* < 0.001 = purple; not significant = yellow.

Figure 1A,B show the normalized high-frequency capacitance (64 kHz) of basal bronchial epithelial cells from control and asthmatic donors, respectively, to investigate how different RBM ECM proteins influence cell attachment. Lower capacitance values over time indicate faster and more complete electrode coverage by cells [35,40]. As shown in Figure 1A,B, basal bronchial epithelial cells attached the fastest on collagen-I (blue), followed by collagen-IV (lilac), collagen-III (green) and fibronectin (red), compared to laminin (yellow) and the BSA protein control (turquoise). Using a one-phase decay model fitted to the control and asthma basal bronchial epithelial cell capacitance curves (Figure 1C), the capacitance rate constant (K), indicating faster cell attachment, was significantly greater for cells grown on collagen-IV and fibronectin compared with laminin and BSA, but collagen-I and -III did not reach significance due to variation between donors.

To compare the attachment dynamics of cells from control and asthmatic donors between ECM conditions, we used a GAM model to assess capacitance changes and visualized the Benjamin–Hochberg–FDR-adjusted pairwise comparisons as heatmaps in Figure 1D,E, respectively. The upper triangle (red) represents differences in curve shape (group-by-time interaction), while the lower triangle (blue) reflects differences in curve position. In the control-derived bronchial epithelial cells, the line position and shape for collagen-I was significantly different to collagen-III, -IV, fibronectin, laminin and BSA, indicating more rapid and complete cell attachment. However, in asthmatic-derived cells, while the line position for collagen-I attachment was significantly different to collagen-III, -IV, fibronectin, laminin and BSA, the line shape indicating the speed of attachment for collagen-I was not significantly different to collagen-IV, but was significant compared to all other ECM proteins.

### 3.2. Basal Bronchial Epithelial Cells Derived from Asthmatic Donors Spread More Rapidly on Collagen-I

**Figure 2:**
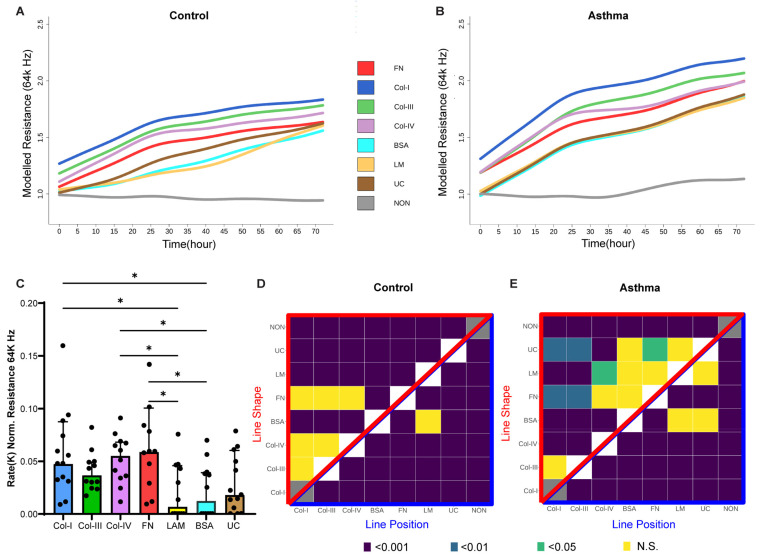
Basal bronchial epithelial cell spreading on different RBM extracellular matrix proteins. Basal bronchial epithelial cells from control (*n* = 6) and asthma (*n* = 7) were grown on ECIS electrodes coated with 50 ug/mL of fibronectin (FN, red), collagen-1(Col-1, blue), collagen-III (Col-III, green), collagen-IV (Col-IV, purple), bovine serum albumin (BSA, turquoise), or laminin (LAM, yellow) and compared to uncoated electrodes (UC, brown) or electrodes with no cells (NC, grey). (**A**,**B**) Generalized additive mixed model (GAMM) curves showing normalized 64 kHz resistance over time for control (**A**) and asthma (**B**) epithelial cells across seven ECM substrates. (**C**) Rate constant (K) was calculated using a one-phase association model; higher values indicate faster spreading. A Kruskal–Wallis test with the two-stage step-up method of Benjamini, Krieger and Yekutieli post hoc was used. (**D**,**E**) Heatmaps of BH-FDR-adjusted *p*-values from pairwise comparisons of GAM-modelled resistance between ECM conditions for control (**D**) and asthma (**E**). The upper triangle (red) represents differences in curve shape (group-by-time interaction), while the lower triangle (blue) reflects average resistance. * *p* < 0.05. *p* < 0.05 = green; *p* < 0.01 = blue; *p* < 0.001 = purple; not significant = yellow.

Figure 2A,B show the normalized high-frequency resistance curves (64 kHz) of basal bronchial epithelial cells from control and asthmatic donors, respectively, to investigate how different RBM ECM proteins influence epithelial cell spreading. A steeper increase in resistance over time indicates more rapid cell spreading [35,40]. As shown in Figure 2A,B, basal bronchial epithelial cells spread the fastest on collagen-I, (blue), followed by collagen-III (green), collagen-IV (lilac), and fibronectin (red), compared to laminin (yellow) and the BSA protein control (turquoise). Using a one-phase association model fitted to the control and asthma basal bronchial epithelial cell capacitance curves (Figure 2C), the rate constant (K) indicated that cell spreading was significantly faster for cells grown on collagen-I, collagen-IV and fibronectin compared with laminin and BSA, but collagen-III did not reach significance due to variation between donors.

To compare the attachment dynamics of cells from control and asthmatic donors between ECM conditions, the pairwise GAM model comparisons over time were visualized as FDR-adjusted heatmaps in Figure 2D,E, respectively. In the control-derived bronchial basal epithelial cells, the line position for collagen-I was significantly different to collagen-III, -IV, fibronectin, laminin and BSA; however, the line shape was only significantly different to laminin and BSA. Notably, in asthmatic-derived cells, the line position for collagen-I attachment was significantly different to collagen-III, -IV, fibronectin, laminin and BSA, and the line shape for collagen-I was significantly different to collagen-IV, fibronectin, laminin and BSA, indicating more rapid and complete cell attachment.

### 3.3. Basal Bronchial Epithelial Cells Derived from Asthmatic Donors Form a Barrier More Rapidly on Collagen-I and Fibronectin

**Figure 3:**
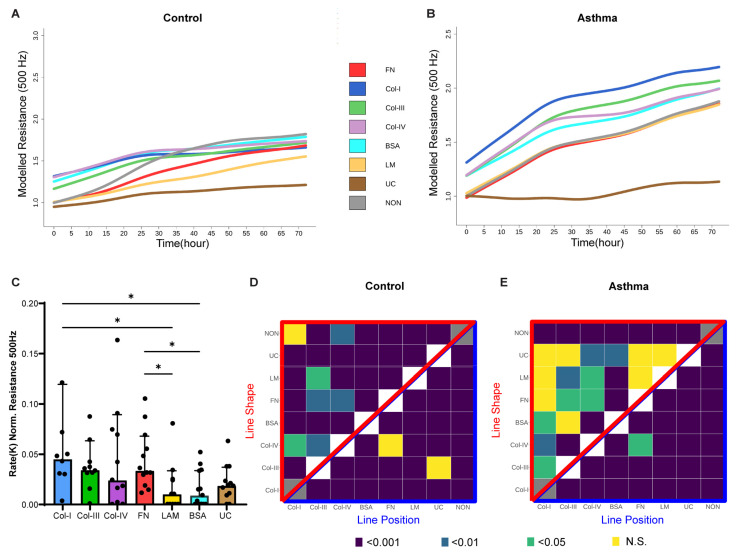
Basal bronchial epithelial cell barrier formation on different RBM extracellular matrix proteins. Basal bronchial epithelial cells from control (*n* = 6) and asthma (*n* = 7) were grown on ECIS electrodes coated with 50 ug/mL of fibronectin (FN, red), collagen-1(Col-1, blue), collagen-III (Col-III, green), collagen-IV (Col-IV, purple), bovine serum albumin (BSA, turquoise), or laminin (LAM, yellow) and compared to uncoated electrodes (UC, brown) or electrodes with no cells (NC, grey). (**A**,**B**) Generalized additive mixed model (GAMM) curves showing normalized 500 Hz resistance over time for control (**A**) and asthma (**B**) epithelial cells across seven ECM substrates. (**C**) Rate constant (K) was calculated using a one-phase association model; higher values indicate faster barrier formation. A Kruskal–Wallis test with the two-stage step-up method of Benjamini, Krieger and Yekutieli post hoc was used. (**D**,**E**) Heatmaps of BH-FDR-adjusted *p*-values from pairwise comparisons of GAM-modelled resistance between ECM conditions for control (**D**) and asthma (**E**). The upper triangle (red) represents differences in curve shape (group-by-time interaction), while the lower triangle (blue) reflects average resistance. * *p* < 0.05. *p* < 0.05 = green; *p* < 0.01 = blue; *p* < 0.001 = purple; not significant = yellow.

Figure 3A,B show the normalized low-frequency resistance curves (500 kHz) of basal bronchial epithelial cells from control and asthmatic donors, respectively, to investigate how different RBM ECM proteins influence epithelial cell tight junction formation. A steeper increase in resistance over time indicates more rapid tight junction formation [35,40]. As shown in Figure 3A,B, basal bronchial epithelial cells formed barriers the fastest on collagen-I, (blue) and collagen-IV (lilac), followed by collagen-III (green), and fibronectin (red), compared to laminin (yellow) and the BSA protein control (turquoise). Using a one-phase association model fitted to the control and asthma basal bronchial epithelial cell capacitance curves (Figure 3C), the rate constant (K) indicated that cell tight junction formation was significantly faster for cells grown on collagen-I and fibronectin compared with laminin and BSA, but collagen-III and -IV did not reach significance due to variation between donors.

To compare the barrier function of cells from control and asthmatic donors between ECM conditions, the pairwise GAM model comparisons over time were visualized as FDR-adjusted heatmaps in Figure 3D,E, respectively. The upper triangle (red) represents differences in curve shape (group-by-time interaction), while the lower triangle (blue) reflects differences in curve position. In the control-derived bronchial basal epithelial cells, the line position and shape for collagen-I was significantly different to collagen-III, -IV, fibronectin, laminin and BSA. In asthmatic-derived cells, the line position for collagen-I attachment was significantly different compared to collagen-III, -IV, fibronectin, laminin and BSA, but the line shape for collagen-I was only significantly different to collagen-III, -IV and BSA.

### 3.4. Comparison Between Control and Asthma Basal Bronchial Epithelial Cells

**Figure 4:**
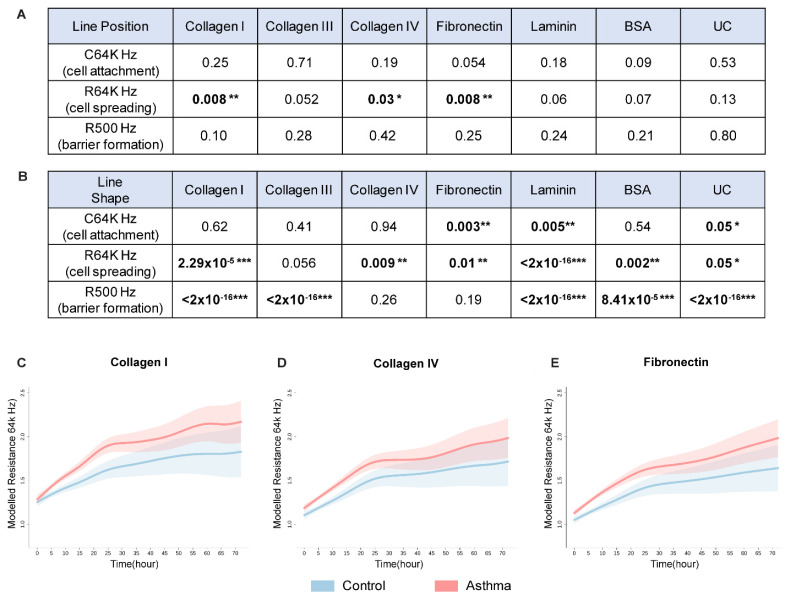
Control and asthma basal bronchial epithelial cell attachment, spreading, and barrier formation on different RBM extracellular matrix proteins. (**A**) Table summarizing curve position metrics (including resistance and capacitance values) for control (*n* = 6) and asthma (*n* = 7) epithelial cells across ECM coatings. (**B**) Table summarizing curve shape metrics obtained from generalized additive mixed effect modelling (GAMM). (**C**–**E**) Representative normalized resistance curves at 64 kHz over time for cells cultured on collagen-I, collagen-IV, and fibronectin. The bold line represents the regression estimate and the shading denotes the standard error of the estimate. * *p* < 0.05, ** *p* < 0.01, *** *p* < 0.001.

To directly compare differences in basal bronchial epithelial cell attachment, spreading and barrier function over time between asthmatic and control donors, we applied a GAM model, incorporating a random effect for biological replicates and smooth time-dependent effects, with a group-by-time interaction. As shown in Figure 4A,B, in terms of cell attachment measured using the normalized high-frequency capacitance (64 k Hz), basal bronchial epithelial cells from individuals with asthma and control did not vary between groups, although in terms of line shape, epithelial cells from asthma donors attached better than control cells on fibronectin (*p* = 0.003) and laminin (*p* = 0.005). As shown in Figure 4A, in terms of cell spreading measured using the normalized high-frequency resistance (64 K Hz), basal bronchial epithelial cells derived from individuals with asthma spread faster (higher position and steeper slope of the curve) on collagen-I (*p* = 0.0008; Figure 4C), collagen-IV (*p* = 0.03; Figure 4D), and fibronectin (*p* = 0.008; Figure 4E) compared to control-derived epithelial cells. In terms of barrier formation, as measured by the normalized low-frequency resistance (500 Hz), basal bronchial epithelial cells derived from control and asthma donors did not vary by line position across ECM proteins, but they did in terms of line shape for collagen-I, -III and laminin.

### 3.5. RBM ECM Proteins Influence Epithelial Cytokine Release

**Figure 5:**
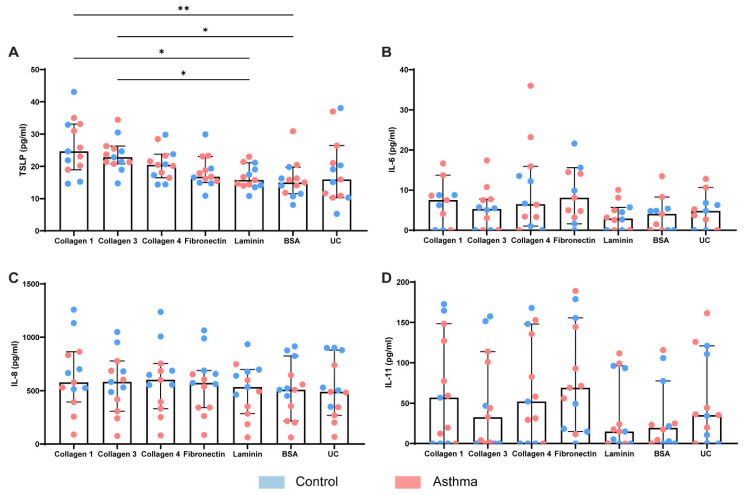
ECM proteins influence the release of epithelial cytokines in primary epithelial bronchial cells. ELISA quantification of cytokines from cell-free supernatants of basal bronchial epithelial cells from control (*n* = 6) and asthma (*n* = 7) grown on various ECM substrates. (**A**) TSLP secretion was significantly increased on collagen-I and collagen-III compared to laminin and BSA. No significant differences were observed across ECM conditions for (**B**) IL-6, (**C**) IL-8, or (**D**) IL-11. Individual data points are colour-coded (blue = control; red = asthma). Data are presented as pg/mL; median with 95% confidence interval; ** *p* < 0.01 and * *p* < 0.05 by Kruskal–Wallis with Dunn’s post hoc test.

To determine whether ECM composition alters epithelial cytokine secretion, we measured cytokine levels in the cell-cultured supernatants collected at 48 h of the ECIS experiment using ELISA. Cytokine responses did not differ between control and asthma bronchial epithelial cells across the different ECM conditions; therefore, datasets were pooled for cytokine analysis. Among the cytokines tested (TSLP, IL-6, IL-8, and IL-11), only TSLP secretion was significantly altered in response to ECM exposure. Specifically, bronchial basal epithelial cells cultured on collagen-I and collagen-III secreted significantly higher levels of TSLP compared to laminin and BSA control (Figure 5A). In contrast, the levels of acute inflammatory proteins IL-6, IL-8, or the fibrotic cytokine IL-11 were not altered by ECM substrates (Figure 5B–D). Cell viability was confirmed across all ECM conditions, with LDH release remaining unaltered and below the positive control in both control and asthma-derived cells (Supplementary Figure S1).

## 4. Discussion

Despite extensive characterization and measurement of reticular basement membrane thickening in asthma, the functional consequences of ECM compositional changes on epithelial behaviour remain poorly defined. This study demonstrates that the composition of the reticular basement membrane ECM exerts a functional influence on primary bronchial basal epithelial cell behaviour. Specifically, fibrillar collagen-I, which is upregulated within the reticular basement membrane of asthmatic airways, promoted more rapid epithelial attachment, spreading, and barrier formation compared with collagen-III, -IV, fibronectin and laminin in both control and asthmatic-derived epithelial cells. In addition, collagen-I and -III promoted increased TSLP secretion from control and asthmatic epithelial cells, further demonstrating that the reticular basement membrane ECM environment can selectively influence epithelial inflammatory responses. Collectively, these data support the concept that alterations in ECM composition due to RBM remodelling in the asthmatic lung may enhance basal epithelial cell attachment and spreading, but also modulates epithelial alarmin release, relevant to type 2 inflammatory signalling in asthma.

The fundamental function of the airway epithelium is to establish an effective mucosal barrier, which depends on the coordinated processes of cell attachment, spreading, barrier formation and differentiation, on an underlying RBM. Among these processes, cell attachment represents a critical initial step required for subsequent cell survival, proliferation and mucosal barrier development. Using electrical cell–substrate impedance sensing (ECIS) to monitor primary human airway epithelial cells from control and asthmatic donors, we demonstrate that the ECM composition characteristic of a remodelled RBM markedly accelerates epithelial attachment dynamics. Specifically, cells exhibited more rapid attachment to fibrillar collagen-I, a key component enriched in the asthmatic RBM compared with the normal airway [29,32,33]. While the role of collagen-IV over laminin in mediating epithelial attachment in the native RBM has been studied extensively, relatively few studies have focused on ECM proteins associated with airway remodelling. Hamilton et al. previously assessed the attachment of primary epithelial cells to tissue culture plates coated with collagen-I, collagen-IV, fibronectin, vitronectin, and laminin within 30 min using fluorescence cell counting to evaluate epithelial repair [41]. While the authors mainly reported that collagen-IV has greater epithelial attachment than laminin, in their assay, fibronectin and collagen-I also demonstrated enhanced cell attachment in line with collagen-IV, compared to laminin at 30 min [41]. Our findings extend these observations by capturing the full temporal dynamics of epithelial attachment using ECIS over 72 h. This distinction is critical, as epithelial adhesion occurs through sequential phases, including initial contact, cell flattening, and cytoskeletal reorganization leading to stable attachment [42], which occurs over hours rather than minutes. In primary airway epithelial cells, the half-life for electrode coverage took hours and was shortest for collagen-I (7.1 h) and fibronectin (7.9 h), followed by collagen-IV (8.6 h) and collagen-III (10.5 h). In contrast, laminin and the BSA control supported slower attachment kinetics with the longest half-lives of 22.9 h and 29.1 h, respectively. Thus, short-term assays may underestimate the functional impact of ECM composition on cell attachment, flattening and cytoskeletal organization that are required for effective cell attachment.

Using the same ECIS platform, we have previously published on epithelial cell attachment dynamics using the 1HAEo- bronchial epithelial cell line, in which fibronectin followed by collagen-I had the fastest epithelial attachment with half-lives of 6.5 and 7.2 h respectively [39]. In contrast, 1HAEo- cell attachment to collagen-IV (11.3 h) was similar to laminin (13.2 h) and the BSA-coated (12.4 h) and uncoated (13.9 h) controls. The hierarchy of ECM-dependent attachment in 1HAEo- cells is broadly consistent with our current primary basal cell findings, where collagen-I and fibronectin again emerged as the substrates with fastest epithelial attachment, though collagen-IV is notably slower than for primary bronchial epithelial cells. This may reflect fundamental intrinsic differences between primary cells and the 1HAEo^−^ cell line, which is SV40-transformed to drive proliferation, and highlights the importance of using primary airway epithelial cells, especially from patients with disease. In terms of basal epithelial attachment, we found no difference in control and asthmatic epithelial cells; for both groups, collagen-I induced the most rapid attachment, followed by fibronectin, collagen-IV, and collagen-III, with similar responses observed between control and asthmatic epithelial cells.

Following initial cell attachment, basal bronchial epithelial cells spread most rapidly when cultured on collagen-I, -IV, and fibronectin substrates, indicating that these ECM proteins promote efficient cell coverage during epithelial repair. Although no prior studies have directly quantified epithelial spreading dynamics using real-time approaches such as ECIS, our findings are supported by the previous work of Mereness et al. [43], who demonstrated that both primary human lung epithelial cells and the 16HBE SV40-transformed bronchial epithelial cell line exhibit enhanced wound closure following a scratch injury when cultured on collagen-I and -VI compared to Matrigel, which is composed of laminin (~60%), collagen IV (~30%), and entactin/nidogen (~8%) and represents a prototypical basement membrane environment. Specifically, scratch wounds remained more than 6-fold larger on Matrigel compared to collagen-I- and -VI-coated substrates, highlighting that a healthy basement membrane environment may promote less epithelial repair than fibrillar collagens. In further support of our findings, collagen-I has been shown to promote airway smooth muscle proliferation, and fibronectin has been shown to enhance epithelial proliferation and survival in the bronchial epithelial cell lines BEAS-2B and 16HBE [44,45]. In terms of a mechanism for how ECM proteins influence cell spreading, Hirst et al. have previously proposed that collagen-I may increase proliferation through the upregulation of the nuclear proliferation marker Ki67 in smooth muscle [44]. While we did not assess Ki67 expression directly in our study, we have previously confirmed in the 1HAEo^−^ cell line that the measurement of capacitance (64 k Hz) correlates significantly with cell number, and that the capacitance (64 k Hz) increases the greatest when 1HAEo^−^ cells are seeded on collagen-I and fibronectin. Thus, the increase in capacitance likely reflects an increase in cell number. Together, these findings suggest that ECM proteins enriched during airway remodelling in asthma, particularly collagen-I and fibronectin, may promote epithelial spreading and proliferation to facilitate early epithelial repair responses.

Notably, asthmatic epithelial cells demonstrated greater spreading on collagen-I, -IV and fibronectin compared to control epithelial cells, indicating an enhanced responsiveness to a remodelled ECM environment. Lung epithelial cells are known to bind collagen-I and -III via integrin α2β1, collagen-III via integrin α1β1 and α2β1, and fibronectin via integrin α5β1 [46]. While previous studies investigating integrin expression in asthmatic epithelial cells have not reported differences in α1β1 and α2β1 integrins, α5β1, which important for fibronectin, has been shown to be reduced in asthmatic compared to control donors [47,48]. These findings suggest that the enhanced spreading of asthmatic bronchial epithelial cells on fibrillar collagen-I and -III observed in the present study is not likely driven solely by altered integrin expression, activation or downstream signalling. Further, while α2β1-mediated adhesion has been shown to be enhanced by IL-13, the cell culture assay did not involve the addition of cytokines, and thus the differences observed in asthmatic compared to control epithelial cells were intrinsic to the cells. In support of a link between RBM airway remodelling and altered epithelial transcription, Bazan-Socha and colleagues demonstrated the association of 148 genes in bronchial brushings from asthmatic patients with airway remodelling. The most significant genes were involved in cell growth, proliferation, metabolism, junctional protein expression, ECM components, oxidative stress and immune response [49]. These findings suggest that RBM remodelling is associated with an altered epithelial transcriptional profile. However, future transcriptomic and proteomic studies will be required to define the molecular mechanisms underlying these responses in asthmatic compared to healthy control epithelial cells.

Next, we assessed epithelial barrier formation and found that, in basal bronchial epithelial cells, collagen-I and fibronectin significantly reduced the half-life required to establish a confluent epithelial barrier, as measured by low-frequency resistance (500 Hz). Although all ECM substrates ultimately supported the formation of a patent barrier within 45 h, the accelerated barrier kinetics observed on collagen-I, especially in asthmatic epithelial cells, suggest that remodelled RBM environments may promote more rapid epithelial barrier formation following injury. This is particularly relevant in asthma, where epithelial barrier disruption and defective repair are well-documented features of disease pathogenesis. Previous studies have demonstrated impaired barrier integrity in airway epithelial cells derived from individuals with asthma, including reduced transepithelial resistance and altered junctional protein organization (including E-cadherin and ZO-1), even under standardized culture conditions [7,14]. Our findings extend this literature by showing that ECM composition itself is a critical determinant of the kinetics of barrier formation for basal bronchial epithelial cells. In particular, fibrillar ECM proteins enriched in the remodelled asthmatic RBM appear to facilitate more rapid establishment of epithelial continuity, potentially reflecting enhanced cell–matrix adhesion, spreading, and cytoskeletal organization during early repair. Similar alterations in epithelial barrier behaviour have been reported in other airway disease contexts. Studies in basal bronchial epithelial cells from individuals with chronic obstructive pulmonary disease (COPD) have demonstrated delayed or reduced barrier formation associated with disease status and smoking history [50], underscoring the sensitivity of epithelial barrier dynamics to both disease severity and the surrounding microenvironment. In the context of asthma, airway epithelial injury and shedding are well-recognized features of asthma pathogenesis, with bronchial biopsies demonstrating areas of denuded basement membrane [51]. This epithelial fragility is accompanied by increased epithelial apoptosis and expansion of basal cell populations (marked by TP63 and cytokeratin 5), which function as progenitor cells [13,52]. During epithelial repair, basal cells proliferate, migrate and differentiate into ciliated and secretory epithelial lineages to restore barrier integrity. However, in asthma, this repair process is dysregulated, with evidence of increased basal cell numbers [53,54]. In the present study, using the ECIS technique we were only able to investigate the attachment, spreading and barrier formation of basal cells in monolayer culture to RBM ECM proteins. We were unable to assess the effect of these RBM proteins on epithelial differentiation and the formation of secretory and ciliated cell phenotypes. For example, Ito et al. demonstrated in a three-dimensional air–liquid interface model that collagen-IV suppresses MUC5AC mucin expression in primary asthmatic epithelial cells [55]. While outside of the scope of this study, future studies using air–liquid-interface cultures will be required to determine if ECM proteins in the remodelled asthmatic RBM also affect lineage specification and differentiation into mucus-secretory cells and ciliated cells.

Finally, we assessed the inflammatory response of basal bronchial epithelial cells exposed to different RBM ECMs. Previous studies have demonstrated that baseline cytokine secretion from the asthmatic airway epithelium is similar to healthy controls; however, increased IL-6 and IL-8 expression has been shown in asthmatic cells following viral infection or pollution exposure [56]. Among the cytokines assessed in this study, IL-6, IL-8, and IL-11 showed no ECM-dependent variation in release. In contrast, TSLP, an epithelial alarmin, showed clear ECM-dependent differences when cells were cultured on collagen-I and -III, compared to laminin and BSA, regardless of disease status. In support of this observation of ECM proteins influencing cytokine expression, circulating cellular fibronectin has previously been shown to correlate with IL-6 levels within the blood and asthma severity [57]. TSLP is a well-characterized epithelial alarmin that plays a central role in initiating type 2 immune response. It activates dendritic cells and promotes the differentiation of naïve T cells into Th2 cells, leading to the production of IL-4, IL-5, and IL-13. TSLP also acts on type 2 innate lymphoid cells (ILC2), mast cells, basophils, and eosinophils, further amplifying type 2 inflammation [10]. In asthma, elevated TSLP expression has been observed in sputum, serum, and bronchoalveolar lavage fluid, and is associated with disease severity, corticosteroid resistance and mucus hypersecretion [58]. The increased basal TSLP secretion we observed in epithelial cells in response to collagen-I and -III may contribute to chronic Th2 inflammation observed in asthmatic patients in vivo by maintaining a state of “epithelial readiness” or priming. This may help explain why asthmatic airways often demonstrate heightened sensitivity to environmental triggers and sustained eosinophilic inflammation even in the absence of active infection or allergen exposure. More recent biologic therapies for asthma such as tezepelumab have targeted TSLP. Clinical trials, including NAVIGATOR and PATHWAY, have demonstrated that tezepelumab significantly reduces asthma exacerbations, improves lung function, and decreases airway eosinophilia, particularly in patients with corticosteroid-resistant disease [59,60]. Our in vitro findings may provide a mechanistic link for the effect of airway remodelling on driving TSLP expression and why continued tezepelumab treatment is ffective in downregulating features of asthma in remodelled airways.

While the use of ECIS enabled high-resolution, real-time assessment of epithelial cell attachment, spreading and barrier formation within a single experiment, there are some limitations to note for this study. The measurements on the array are limited to monolayer experiments whereas the bronchiolar epithelium is pseudostratified, containing both basal, secretory and ciliated cells. However, during epithelial repair the primary progenitor cells are the basal cells and therefore the experiments in this study do represent the initial stages of wound repair, but do not capture the events following barrier formation including the differentiation of basal cells. Second, the study included a small number of primary control (*n* = 6) and asthma-derived (*n* = 7) epithelial cells which were primarily from fatal asthmatic patients, limiting the applicability of our results to patients with mild and moderate disease. However, RBM remodelling has been shown to occur across all disease severities in asthma [38,61], and we have previously demonstrated that the lungs used for epithelial isolation in this study did have remodelling of the RBM [36,37,38]. Third, bronchiolar epithelial cells are limited in their passage potential and therefore the collection and use of primary epithelial cells to assess multiple ECM proteins in duplicate was limited in this study. Fourth, we could only recover 1 mL of cell culture media for each ECM protein, which limited the number of ELISAs that could be performed in duplicate. A future larger screen would be useful to assess other epithelial DAMPs such as IL-33 and IL-1α or growth factors associated with airway remodelling such as transforming growth factor β. Lastly, the ECM proteins available for cell culture were derived from different species sources, which may introduce variability in protein structure and integrin-binding properties, despite being well used in the literature for human cell culture experiments.

## 5. Conclusions

In summary, this study demonstrates that ECM composition characteristic of the remodelled RBM in asthma is an important determinant of airway epithelial cell behaviour. Specifically, increased deposition of collagen-I, -III, and fibronectin, hallmarks of RBM remodelling, promotes more rapid epithelial attachment, spreading, and barrier formation, suggesting a potential role in facilitating early epithelial repair. However, collagen-I and -III also selectively enhance TSLP secretion, indicating that they may simultaneously contribute to the maintenance of chronic type 2 inflammation within the asthmatic lung. These findings support a model in which RBM remodelling is not solely a structural consequence of asthma but a functionally active microenvironment that modulates epithelial repair and inflammatory signalling. Thus, while airway remodelling may occur to enhance epithelial repair responses, it also results in persistent inflammation within the asthmatic airways.

## Figures and Tables

**Table 1:** Patient demographics. Values given are the means (standard deviation) [SD] or *n* (%), as appropriate. A one-way ANOVA with Tukey’s pairwise comparison showed no significant differences in sex, age, height, weight and smoking history between all groups. SABA = short acting beta-2 adrenergic agonist; LABA = long-acting beta-2 adrenergic agonist; ICS = inhaled corticosteroid; OCS = oral corticosteroid. None of the patients included in this study received biologics or other genetically engineered therapies.

	Control	Asthma
**N**	6	7
Average age (SD)	15.2 (8.5)	14.1 (4.1)
% female	33.3	42.9
Non-Fatal: Fatal	-	1:6
Height (SD) [cm]	160.3 (30.9)	164.3 (13.9)
Weight (SD) [kg]	57.3 (31.6)	56.7 (18.1)
Ethnicity/race (%)		
White	66.7	85.7
Others	33.3	14.3
Medications (%)		
ICS	-	42.9
SABA	-	71.4
LABA	-	28.6
Anti-leukotriene	-	28.6
Anti-histamine	-	42.9
Smoking History [pack years]	-	0.1 (0.4)

## Data Availability

The raw data supporting the conclusions of this article will be made available by the authors upon request.

## Supplementary Materials

The following supporting information can be downloaded at: https://www.mdpi.com/article/10.3390/arm94030038/s1, Figure S1: Lactate dehydrogenase (LDH) release from basal bronchial epithelial cells cultured on extracellular matrix substrates.

## Abbreviations

The following abbreviations are used in this manuscript:

ANOVA	Analysis of Variance
BEC	Bronchial Epithelial Cell
BSA	Bovine Serum Albumin
DAMP	Damage-Associated Molecular Pattern
ECIS	Electric Cell Impedance Sensing
ECM	Extracellular Matrix
GAMM	Generalized Additive Mixed Model
IL	Interleukin
LDH	Lactate Dehydrogenase
RBM	Reticular Basement Membrane
TSLP	Thymic Stromal Lymphopoietin
