# Supplementary material for: Reticular Basement Membrane Remodelling Regulates Bronchial Epithelial Attachment, Barrier Integrity and Inflammatory Signalling in Asthma
Source: Adv Respir Med. 2026 Jun 10;94(3):38. doi: 10.3390/arm94030038 (PMC13295410; doi:10.3390/arm94030038)
Supplement: Supplementary file 1 [file arm-94-00038-s001.zip › arm-4324279-supplementary.pdf]

ONLINE DATA SUPPLEMENT

Reticular Basement Membrane Remodelling Regulates Bronchial Epithelial Attachment, Barrier Integrity and Inflammatory Signalling in Asthma

Aileen Hsieh, Jenna Barker-Mulleder, Chen Xi Yang, May Fouadi, and Tillie Hackett

Supplemental Figure

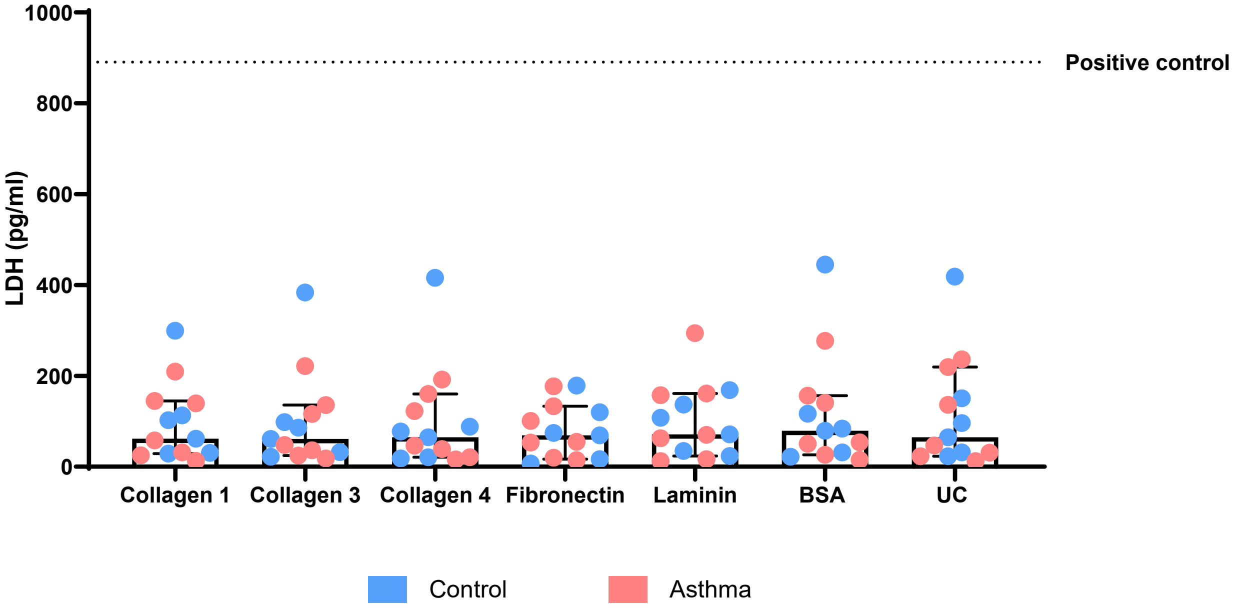

Figure S1. Lactate dehydrogenase (LDH) release from basal bronchial epithelial cells cultured on extracellular matrix substrates. Cell-free supernatants were collected at 48 hours from basal bronchial epithelial cells from control(n=6) and asthma (n=7) cultured on ECIS electrodes coated with collagen I, collagen III, collagen IV, fibronectin, laminin or bovine serum albumin (BSA), or left uncoated (UC). LDH concentration (pg/mL) was quantified as an index of cell death. The dotted line indicates the positive control (maximum LDH release). Cell viability was maintained across all conditions in control and asthma. Individual data points are colour-coded (blue = control, red = asthma). Data are presented as pg/mL; median with 95% confidence interval. No significant differences in LDH release were detected across ECM substrates or between disease groups.
